# Associations between eHealth literacy and 24-hour movement behaviors in older adults: the mediating and moderating roles of self-efficacy

**DOI:** 10.3389/fmed.2026.1746861

**Published:** 2026-03-11

**Authors:** Ning Su, Jiayu Hu, Borui Shang, Xiang Wang, Wei Liang, Lin Zhou, Hao Liu

**Affiliations:** 1 School of Physical Education, Shenzhen University, Shenzhen, China; 2 Department of Social Sciences, Hebei Sport University, Shijiazhuang, China; 3 Department of Sport, Physical Education and Health, Hong Kong Baptist University, Hong Kong, Hong Kong SAR, China; 4 College of Sport and Health, Shandong Sport University, Jinan, China

**Keywords:** 24-hour movement behaviors, eHealth literacy, healthy aging, older adults, self-efficacy

## Abstract

**Background:**

Older adults increasingly rely on digital health resources, yet evidence regarding the relationship between eHealth literacy (eHL) and 24-hour movement behaviors (24-HMB), including physical activity (PA), sedentary behavior (SB), and sleep, remains underexplored. This study examined the associations between eHL and 24-HMB in Chinese older adults and examined self-efficacy as a potential mediator and moderator.

**Methods:**

Using a convenience sampling approach, 564 community-dwelling older adults (aged 60–74 years) were recruited from four urban Chinese cities via an online survey. A total of 553 valid cases were retained for analyses. eHL was assessed using the eHealth Literacy Scale–Web 3.0, and self-efficacy was assessed using a validated Self-Efficacy Scale. PA and SB were assessed objectively using ActiGraph GT3X+ accelerometers over three consecutive days (two weekdays and one weekend day). Sleep duration was derived from accelerometer-based estimates anchored by daily sleep logs. Multiple linear regression analyses were conducted to examine associations, and mediation and moderation were tested using PROCESS macro (Model 4 and Model 1, respectively), adjusting for age, sex, and education.

**Results:**

After adjustment for covariates (*n* = 553), higher eHL was associated with greater light physical activity (LPA) (*b* = 0.43, *p* < 0.001) and moderate-to-vigorous physical activity (MVPA) (*b* = 0.19, *p* < 0.001) as well as lower SB (*b* = −0.43, *p* < 0.001). eHL was also positively associated with self-efficacy (*b* = 0.47, *p* < 0.001). Mediation analyses showed small but statistically significant indirect effects via self-efficacy for LPA [0.04, 95% CI (0.00, 0.09)], MVPA [0.08, 95% CI (0.03, 0.13)], and SB [−0.06, 95% CI (−0.10, −0.02)], whereas no mediation was observed for sleep duration [0.02, 95% CI (−0.03, 0.07)]. Moderation analyses showed significant interactions for LPA (*F* = 76.82, *p* < 0.001), SB (*F* = 75.07, *p* < 0.001), and sleep duration (*F* = 4.74, *p* < 0.001), but not MVPA. Interaction effects for sleep were small in magnitude and should therefore be interpreted with caution. Mediation models explained 26% of the variance in LPA, 6% in MVPA, and 30% in SB.

**Conclusion:**

In this cross-sectional, urban, device-using sample of older adults, higher eHL was associated with a more favorable 24-HMB profile, particularly higher LPA and lower SB, while associations with sleep duration were weaker. Self-efficacy showed modest indirect associations consistent with partial mediation for PA and SB and also acted as a moderator of several associations. Given the observational design and modest effect sizes, findings should be interpreted cautiously and require confirmation in longitudinal or experimental studies with more representative sampling and improved sleep assessment.

## 1 Introduction

China is facing an accelerating demographic shift toward population aging. By 2022, more than 280 million Chinese residents were aged ≥60 years (19.8% of the total population), and this proportion continues to rise (1). Population aging imposes substantial pressures on socioeconomic systems while placing higher demands on health management for older adults. Promoting healthy aging and improving quality of life among older Chinese adults has therefore become a pressing public health priority. Within this context, 24-hour movement behaviors (24-HMB), comprising physical activity (PA), sedentary behavior (SB), and sleep, have emerged as key determinants of healthy aging (2). Research consistently shows that more favorable 24-HMB profiles in older adults are associated with reduced risks of chronic disease, improved functional capacity, and better cognitive and mental health (3–6). However, due to age-related physiological decline, reduced social participation, entrenched lifestyle habits, and limited access to information, many older Chinese adults struggle to maintain healthy 24-HMB patterns, posing challenges for health management.

The rapid proliferation of the internet and mobile technologies has created new opportunities for older adults to manage their health via electronic health (eHealth). eHealth enables individuals to access health information, monitor behaviors, and communicate with healthcare professionals through digital platforms (7, 8). These benefits, however, depend on adequate eHealth literacy (eHL), defined as the ability to search for, understand, evaluate, and apply eHealth information to address health problems or make health-related decisions (9, 10). Although higher eHL has been linked to positive health behaviors and outcomes in older populations (8, 11), evidence regarding its relationship with the integrated framework of 24-HMB remains limited. Moreover, little is known about the mechanisms through which eHL may influence PA, SB, and sleep collectively. Beyond information access, eHL may be linked to movement behaviors by supporting self-regulation (e.g., goal setting, planning, and self-monitoring) via digital tools. However, empirical evidence remains limited among older adults within an integrated 24-HMB framework, particularly when using objectively assessed behaviors and theory-driven tests of indirect associations and moderating effects. Notably, existing evidence is limited in several respects. First, most studies have examined eHL in relation to single health behaviors or healthcare utilization, rather than jointly considering PA, SB, and sleep within an integrated 24-HMB framework. Second, prior research has often been predominantly descriptive, with limited theory-driven testing of the psychological mechanisms that may link eHL to movement-behavior patterns. Third, evidence among older adults, particularly in rapidly aging and digitally transforming contexts such as China, remains scarce. Addressing these gaps, the present study draws on Social Cognitive Theory (SCT) to examine whether self-efficacy helps explain (mediates) and shapes (moderates) the associations between eHL and 24-HMB.

SCT offers a valuable lens for understanding the formation and change of health behaviors (12). SCT posits dynamic, reciprocal interactions among behavior, personal factors (e.g., cognitive, affective, and biological events), and environmental influences, emphasizing that behavior emerges from continuous person-environment-behavior interplay rather than environmental determinants alone (13). Within SCT, self-efficacy, the belief in one’s capability to perform a specific behavior in a given context, is a key predictor and explanatory factor for health behavior (14). Higher self-efficacy enhances resilience in the face of challenges, promotes goal setting, and fosters persistence, thereby facilitating behavior maintenance and change (15). In China, older adults’ self-efficacy may vary with traditional beliefs, family support, and social contexts, highlighting the importance of considering self-efficacy when examining the relationship between eHL and 24-HMB.

Guided by SCT, self-efficacy may function in two complementary ways. First, it may mediate the relationship between eHL and 24-HMB, such that older adults with higher eHL gain greater confidence in their ability to adopt healthier patterns by understanding and applying digital health information. Second, it may moderate this relationship, strengthening the behavioral impact of eHL among those with higher self-efficacy. Importantly, mediation and moderation were examined in separate models as complementary questions, rather than being modeled simultaneously within a single causal model.

It is also important to acknowledge that older adults’ engagement in movement behaviors and eHealth use may be shaped by broader confounding factors, such as demographic characteristics, health conditions, functional limitations, and prior digital experience, as documented in previous literature (16–18). In particular, demographic characteristics including age, sex, and educational attainment are consistently identified as correlates of eHL and movement behaviors in older adults (19, 20), highlighting that these covariates should be considered when examining associations between eHL and movement behaviors.

In addition, although movement behaviors can be domain-specific, the present study assessed self-efficacy using a validated 5-item scale measuring general self-efficacy for health behavior change. This choice is theoretically consistent with the integrated nature of the 24-HMB framework, which conceptualizes PA, SB, and sleep as interdependent components of a unified 24-hour behavior cycle (2). Accordingly, older adults often adopt changes in these behaviors simultaneously rather than in isolation, making a domain-general measure of confidence in engaging in lifestyle modifications appropriate for capturing the overarching psychological mechanism through which eHL may influence multiple behaviors (2, 21, 22). Importantly, the scale includes items that explicitly reference improvements in PA, reductions in SB, and the adoption of healthy sleep routines, ensuring relevance to each behavioral domain. Prior research has also demonstrated strong psychometric properties of this scale among Chinese older adults (23), further supporting its suitability for examining the mediating and moderating mechanisms linking eHL to daily movement behaviors.

Therefore, this study aimed to examine associations between eHL and the 24-HMB in older Chinese adults and to investigate the mediating and moderating roles of self-efficacy. Based on SCT and prior evidence, we formulated the following hypotheses:

H1: Higher eHL is associated with more favorable 24-HMB (higher PA, lower SB, and adequate sleep duration).

H2: Self-efficacy mediates the relationships between eHL and each movement behavior.

H3: Self-efficacy moderates the associations between eHL and 24-HMB, such that the relationships are stronger among individuals with higher self-efficacy.

By clarifying these pathways, this study seeks to inform the development and refinement of eHealth-related strategies that may enhance both eHL and self-efficacy, ultimately supporting healthy aging through a more age-friendly digital health environment and stronger self-management among older adults. However, longitudinal or experimental research is needed to establish temporal ordering and causal effects.

## 2 Methods

### 2.1 Participants

Using a convenience sampling approach, community-dwelling older adults were recruited from four urban cities in China that were comparable in overall economic development, urban infrastructure, and access to digital health resources, as indicated by publicly available socioeconomic indicators (e.g., GDP per capita and digital infrastructure coverage). Recruitment was conducted between March and June 2023 through community centers, local senior activity groups, and online community platforms.

Given the eHealth focus and online survey format, the analytic sample primarily represents urban, community-dwelling, device-using older adults aged 60–74 years. City selection aimed to reduce contextual heterogeneity in digital health resource access and was not intended to address selection bias.

Eligibility criteria included: (a) aged 60–74 years; (b) without mobility impairments; (c) without diagnosed cognitive impairment; (d) able to read and understand Chinese; and (e) able to use a smartphone or computer independently. These criteria were adopted to ensure participants could complete the online survey and interact with digital health-related content, which was central to the study focus. The upper age limit (74 years) was set to enhance feasibility and data quality by reducing the likelihood of substantial cognitive or functional impairment that could compromise online survey completion and accelerometer compliance.

A total of 564 older adults initially responded to the survey invitation. After excluding individuals with substantial missing data, inconsistent response patterns, or failure to meet eligibility criteria, 553 valid cases were retained for analysis, yielding a valid response rate of 98.05%. Individuals who were unable to use digital devices or declined participation during the screening stage were not included, which may limit the generalizability of the findings to older adults with basic digital access and literacy.

### 2.2 Procedures

Eligible participants completed an online informed consent form prior to participation. After consent, participants completed a structured online questionnaire assessing sociodemographic characteristics, eHL, and self-efficacy. Subsequently, participants were invited to wear an ActiGraph GT3X+ accelerometer on the right hip for three consecutive days (two weekdays and one weekend day) during waking hours and to remove it only for bathing or swimming. Standardized written instructions and a wear-time log were provided, and research staff sent daily text messages or phone reminders to promote adherence.

All study procedures were conducted in accordance with the Declaration of Helsinki and approved by the Medical Ethics Committee of the Faculty of Medicine at Shenzhen University (Approval No. PN-202300066). All data were de-identified and stored in encrypted files accessible only to authorized researchers.

### 2.3 Measures

#### 2.3.1 Demographic characteristics

Data on age, sex, marital status, educational attainment, living arrangement (cohabiting vs. living alone), and body mass index (BMI) were collected.

#### 2.3.2 eHealth literacy

eHL was assessed using the eHealth Literacy Scale-Web 3.0 developed for Chinese adults by Liu et al. (24). The instrument comprises 24 items across three dimensions (i.e., acquisition, verification, and application), rated on a 5-point Likert scale (1 = strongly disagree to 5 = strongly agree), with higher total scores indicating greater eHL. This instrument captures participants’ self-reported (perceived) competencies in acquiring, verifying, and applying online health information, rather than objectively tested performance. Importantly, this scale assesses not only advanced digital skills but also basic capacities related to accessing, evaluating, and applying online health information, making it suitable for older adults with varying levels of digital exposure. In the present study, participants were required to have basic access to and familiarity with digital devices, which is consistent with the intended scope of the scale. The reliability and validity of the scale in Chinese older adults have been supported [Cronbach’s *α* = 0.93; *χ^2^/df* = 2.722; CFI = 0.955; TLI = 0.948; RMSEA (95% CI) = 0.051 (0.047, 0.056); SRMR = 0.032] (11).

#### 2.3.3 Self-efficacy

Self-efficacy was measured using a 5-item scale adapted from previous research (23). Each item began with the stem “I am confident that….” A sample item is “…I can adopt a healthy lifestyle, including engaging in sufficient physical activity, reducing sedentary time, and maintaining adequate and regular sleep.” Items were rated on a 5-point Likert scale (1 = strongly disagree to 5 = strongly agree), with higher scores indicating greater self-efficacy for health behavior change. In this study, the scale was used to operationalize domain-general lifestyle self-efficacy for adopting integrated health behavior changes across PA, SB, and sleep, rather than behavior-specific efficacy for each outcome. Although PA, SB, and sleep are behaviorally distinct, the present study focused on self-efficacy as a domain-general psychological resource reflecting confidence in adopting integrated lifestyle modifications. This approach is consistent with the 24-HMB framework, which emphasizes the interdependence of these behaviors across the day. The scale items explicitly reference confidence related to multiple behavioral domains, allowing it to capture the overarching self-regulatory belief underlying changes across PA, SB, and sleep. The scale demonstrated satisfactory psychometric properties among Chinese older adults [Cronbach’s *α* = 0.87; *χ^2^/df* = 4.787; CFI = 0.983; TLI = 0.956; RMSEA (95% CI) = 0.056 (0.018, 0.097); SRMR = 0.016] (23).

#### 2.3.4 Health behaviors

PA and SB were objectively assessed using an ActiGraph GT3X+ accelerometer (Pensacola, FL, USA), worn on the right hip for three consecutive days (two weekdays and one weekend day). Participants were instructed to wear the device during all waking hours, removing it only for swimming and bathing, and to maintain their usual routines. Data were sampled at 30 Hz and aggregated into 60-s epochs. Non-wear time was defined as ≥60 consecutive minutes of zero counts, allowing for interruptions of up to 2 min with counts below 100 counts/min, and was excluded from analysis. This commonly used algorithm may still misclassify some prolonged sedentary bouts as non-wear; therefore, sedentary time estimates should be interpreted cautiously. A valid day was defined as ≥10 h of wear time. Although longer monitoring periods are generally recommended to better estimate habitual activity, a 3-day protocol including two weekdays and one weekend day has been used in community-based studies of older adults and may capture meaningful variation in daily movement behavior while minimizing participant burden (25, 26). However, a 3-day protocol may provide less stable estimates of habitual PA and SB than longer monitoring (e.g., 7 days), and results should be interpreted accordingly. Activity intensity was classified using established cut-points proposed by Miller et al. (27), with light physical activity (LPA) defined as 100–1951 counts/min, moderate-to-vigorous physical activity (MVPA) as ≥1952 counts/min, and SB as <100 counts/min. Although these cut-points were originally developed in adult samples, Miller et al. (27) included older adults aged 60–69 years and demonstrated that accelerometer-derived estimates of absolute PA intensity were comparable across age groups. These thresholds have also been widely applied in studies involving older populations (28, 29). Notably, recent evidence syntheses have highlighted that device-based assessments of movement behaviors in older adults are characterized by greater uncertainty and heterogeneity, particularly for SB outcomes, underscoring the importance of transparent reporting and cautious interpretation of accelerometry protocols in this population (30).

Sleep duration was assessed as part of the 24-HMB. During the accelerometer wear period, participants completed daily sleep logs reporting bedtime and wake time. These self-reported sleep logs were used to assist in identifying nocturnal sleep periods and to anchor the bedtime–wake-time window for deriving sleep duration estimates from the accelerometer. Sleep duration was calculated based on the nocturnal sleep period anchored by the sleep log-reported bedtime and wake time and was averaged across valid monitoring nights. Accelerometer-based estimates were used as the primary measure of sleep duration in the analyses, acknowledging that hip-worn accelerometry provides an indirect measure of sleep and should be interpreted cautiously.

### 2.4 Data analysis

All statistical analyses were performed using SPSS version 27.0 (IBM Corp., Armonk, NY, USA). Data were screened prior to analyses. Univariate outliers were identified as values exceeding ±3 standard deviations from the mean and were excluded. After data cleaning and exclusion of invalid responses, the final analytic sample consisted of 553 participants. A sensitivity power analysis (*α* = 0.05, power = 0.80) indicated that with *n* = 553, the regression models could detect small incremental effects (Cohen’s *f*^2^ = 0.014) for one-degree-of-freedom tests in models including multiple covariates. Descriptive statistics were computed for all demographic characteristics and study variables. Given the cross-sectional, individual-level design and continuous outcomes, multiple linear regression and the PROCESS macro were used, with bootstrap resampling to estimate indirect effects for mediation and regression-based interaction terms for moderation. Multiple linear regression models were first used to examine the associations between demographic characteristics (i.e., age, sex, education level, marital status, living arrangement, and BMI) and each 24-HMB outcome (i.e., LPA, MVPA, SB, sleep duration). These analyses were conducted to identify demographic correlates of movement behaviors and to inform the selection of covariates for subsequent mediation and moderation analyses. Associations between eHL, self-efficacy, and 24-HMB were then examined using multiple linear regression models. Prior to model estimation, continuous predictor variables were mean-centered to reduce multicollinearity, particularly for moderation analyses. Regression assumptions, including linearity, normality of residuals, and homoscedasticity, were evaluated through inspection of residual plots and standardized residuals, with no major violations observed. Variance inflation factors (VIFs) were examined to assess multicollinearity, and all VIF values were below commonly accepted thresholds. Mediation analyses were performed using the PROCESS macro (Model 4) with 5,000 bias-corrected bootstrap resamples to test whether self-efficacy mediated the associations between eHL and each movement behavior outcome. Moderation analyses were performed using PROCESS Model 1 to examine whether self-efficacy moderated these associations. Covariates included age, sex, and educational attainment, selected based on theoretical relevance and their observed associations with the outcome variables in the present study. Finally, sensitivity analyses were conducted to assess the robustness of the findings by randomly excluding 5% of participants (resulting in *n* = 525) and re-running the mediation (PROCESS Model 4) and moderation (PROCESS Model 1) models using the same covariates and analytic specifications as in the primary analyses. The direction and statistical significance of key effects (i.e., indirect effects and interaction terms) were compared with those from the full-sample analyses. Statistical significance was set at *p* < 0.05 (two-tailed).

## 3 Results

### 3.1 Descriptive statistics

A total of 553 older adults were included in the analyses. The mean age was 66.28 ± 4.11 years, and 45.6% were men (*n* = 252) and 54.4% were women (*n* = 301). Most participants were married (92.6%) and lived with others (91.3%). Educational attainment was predominantly college-level or above (69.3%). The mean BMI was 22.58 ± 2.54 kg/m^2^, with 65.1% classified as normal weight. The mean scores for eHL and self-efficacy were 3.23 ± 0.57 and 3.59 ± 0.66, respectively. Regarding 24-HMB, the average daily duration was 154.83 ± 120.37 min for LPA, 27.26 ± 25.82 min for MVPA, 669.05 ± 139.53 min for SB, and 448.86 ± 65.81 min for sleep. Daily values of PA, SB, and sleep duration were first averaged across each participant’ valid wear days. Sleep duration values were derived from accelerometer-based estimates anchored by daily sleep logs, as described in the Methods. These participant-level daily averages were then used to compute the sample-level descriptive statistics reported in Table 1. The relatively large variability observed in LPA and SB reflects substantial inter-individual heterogeneity in daily movement patterns among older adults. Prior to analysis, data were screened for extreme values, and univariate outliers exceeding ± 3 standard deviations from the mean were excluded, as described in the Methods. Visual inspection of distributions indicated that PA variables exhibited mild positive skewness, which is common in accelerometer-derived activity data in older populations. These distributional characteristics were considered in subsequent regression analyses. Detailed descriptive characteristics are presented in Table 1. Collectively, these descriptive patterns provide the behavioral context for examining how eHL relates to variability in older adults’ daily movement profiles.

**Table 1:** Descriptive characteristics of the study sample (*n* = 553).

Demographic information	Mean (SD)/frequency (%)
Age	66.28 (4.11)
Gender	
Female	301 (54.4%)
Male	252 (45.6%)
Marital status	
Married	512 (92.6%)
Single, divorced, or widowed	41 (7.4%)
Education level	
Primary and below	45 (8.1%)
Middle and high school	125 (22.6%)
College and above	383 (69.3%)
Living situation	
Living with others	505 (91.3%)
Living alone	48 (8.7%)
BMI category (kg/m^2^)	22.58 (2.54)
Underweight	28 (5.1%)
Normal weight	360 (65.1%)
Overweight and obese	164 (29.7%)
eHL	3.23 (0.57)
Self-efficacy	3.59 (0.66)
PA (min/day)	
LPA (min/day)	154.83 (120.37)
MVPA (min/day)	27.26 (25.82)
SB (min/day)	669.05 (139.53)
Sleep (min/day)	448.86 (65.81)

BMI, body mass index; eHL, eHealth literacy; PA, physical activity; LPA, light physical activity; MVPA, moderate-to-vigorous physical activity; SB, sedentary behavior.

### 3.2 Demographic correlates of PA, SB, and sleep

As shown in Table 2, multiple linear regression analyses indicated that LPA was significantly associated with age (*β* = −0.21, *p* < 0.001), sex (reference: men; *β* = 0.17, *p* < 0.001), and education level (reference: college or above; primary or below *β* = −0.41, *p* = 0.009). SB was significantly associated with age (*β* = 0.19, *p* < 0.001), sex (*β* = −0.15, *p* < 0.001), and education (primary or below *β* = 0.63, p < 0.001). Sleep duration was also associated with education level (primary or below *β* = −0.49, *p* = 0.002; middle/high school *β* = −0.21, *p* = 0.038). In contrast, marital status, living arrangement, and BMI were not significantly associated with PA, SB, or sleep duration in the regression models (*p* = 0.06–0.99), suggesting that variability in movement behaviors was more strongly patterned by age, sex, and educational attainment. MVPA was not significantly associated with age, which may reflect generally low and highly variable MVPA participation among older adults. Taken together, these findings support the inclusion of age, sex, and educational attainment as key covariates in the subsequent mediation and moderation models.

**Table 2:** Associations between demographic characteristics and PA, SB, and sleep.

Variable	LPA (min/day)		MVPA (min/day)		SB (min/day)		Sleep (min/day)	
	*β*	*p* value	*β*	*p* value	*β*	*p* value	*β*	*p* value
Age	−0.21	<0.001	−0.05	0.26	0.19	<0.001	0.001	0.97
Gender (reference: male)	0.17	<0.001	0.04	0.34	−0.15	<0.001	−0.01	0.83
Marital status (reference: married)	0.02	0.67	−0.03	0.49	−0.01	0.89	−0.01	0.81
Education level (reference: college and above)								
Primary and below	−0.41	0.009	−0.25	0.11	0.63	<0.001	−0.49	0.002
Middle and high school	−0.15	0.16	−0.17	0.10	0.26	0.12	−0.21	0.038
Living situation (reference: living with others)	−0.07	0.10	0.08	0.06	0.04	0.34	0.01	0.79
BMI (kg/m^2^)	0.03	0.48	0.02	0.70	<0.001	0.99	−0.06	0.14
BMI category (reference: overweight and obese)								
Underweight	−0.05	0.79	−0.11	0.61	<0.001	0.99	0.15	0.47
Normal weight	−0.09	0.37	−0.01	0.95	0.08	0.41	−0.01	0.91

*β* indicates standard regression coefficients. Table 2 presents exploratory analyses of demographic correlates of 24-hour movement behaviors (24-HMB) to inform covariate selection for subsequent mediation and moderation analyses. BMI, body mass index; PA, physical activity; LPA, light physical activity; MVPA, moderate-to-vigorous physical activity; SB, sedentary behavior.

### 3.3 Associations of eHL with 24-HMB and the mediating role of self-efficacy

**Figure 1:**
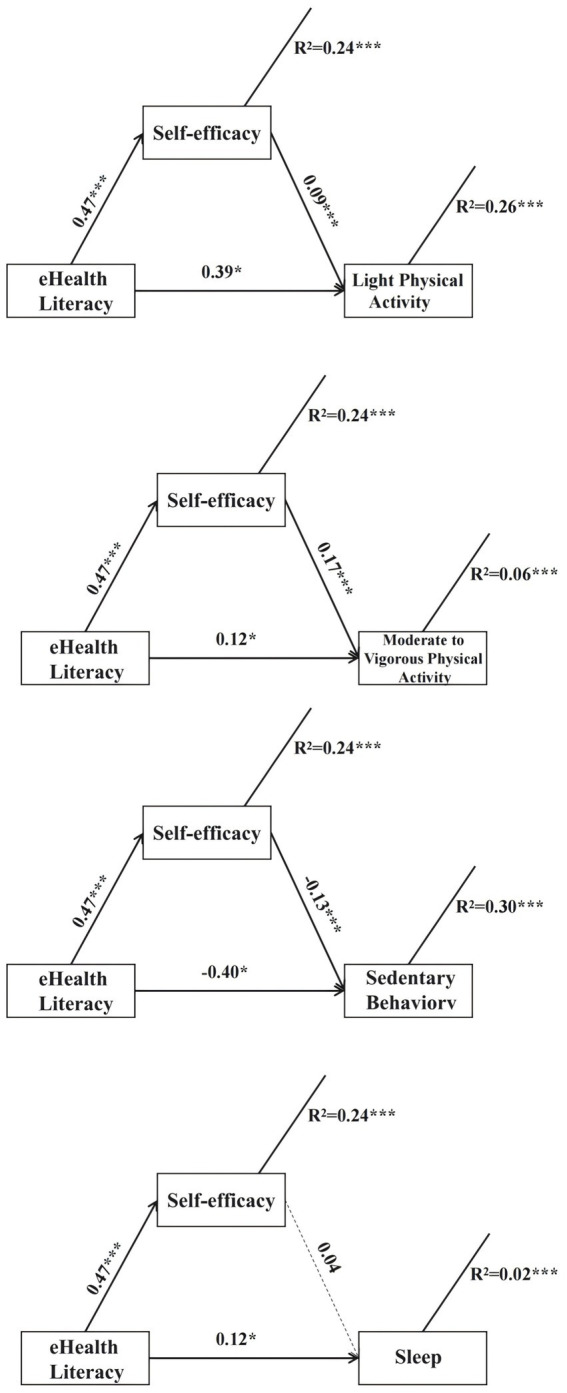
Mediation models illustrating the indirect effects of eHL on LPA, MVPA, SB, and sleep through self-efficacy. Age, sex, and educational level were controlled as covariates in all mediation models. Solid lines represent significant paths, whereas dashed lines represent non-significant paths. Values reported are standardized path coefficients. ^*^p < 0.05, ^**^p < 0.01, ^***^p < 0.001. eHL, eHealth literacy. LPA, light physical activity. MVPA, moderate-to-vigorous physical activity. SB, sedentary behavior.

After adjusting for age, sex, and education, higher eHL was significantly associated with greater LPA (*b* = 0.43, *p* < 0.001) and MVPA (*b* = 0.19, *p* < 0.001), as well as lower SB (*b* = −0.46, *p* < 0.001). eHL was also positively associated with self-efficacy (*b* = 0.47, *p* < 0.001). When self-efficacy was included in the models, it significantly predicted LPA and MVPA and was negatively associated with SB (see Figure 1).

Mediation analyses indicated that self-efficacy partially mediated the associations between eHL and LPA, MVPA, and SB. As shown in Table 3, although the indirect effects were statistically significant, their magnitudes were small [LPA: 0.04, 95% CI (0.00, 0.09); MVPA: 0.08, 95% CI (0.03, 0.13); SB: −0.06, 95% CI (−0.10, −0.02)], indicating modest mediation effects. For sleep duration, the total effect of eHL was statistically significant (*b* = 0.14, *p* < 0.01); however, as shown in Table 3, neither the indirect effect through self-efficacy [0.02, 95% CI (−0.03, 0.07)] nor the overall mediation model reached statistical significance. These results suggest that self-efficacy did not mediate the association between eHL and sleep duration. Overall, the results provide support for modest indirect pathways via self-efficacy for PA and SB, whereas no mediation was observed for sleep duration.

**Table 3:** Total, direct, and indirect effects of eHL on LPA, MVPA, SB, and sleep through self-efficacy (*n* = 553).

Variable	*Est* (95% CI)
Total effect	
eHL → LPA	0.43 ^***^
eHL → MVPA	0.19^***^
eHL → SB	−0.46^***^
eHL → sleep	0.14^**^
Indirect effects	
eHL → self-efficacy → LPA	0.04 (0.00, 0.09)
eHL → self-efficacy → MVPA	0.08 (0.03, 0.13)
eHL → self-efficacy → SB	−0.06 (−0.10, −0.02)
eHL → self-efficacy → sleep	0.02 (−0.03, 0.07)
Direct effect	
eHL → LPA	0.39 ^***^
eHL → MVPA	0.12^*^
eHL → SB	−0.40^***^
eHL → sleep	0.12^*^

Age, sex, and educational level were controlled as covariates in all models; Est represents the estimated standardized effect; 95% CI denotes the 95% confidence interval estimated using bias-corrected bootstrap procedures with 5,000 resamples; *p < 0.05, **p < 0.01, ***p < 0.001. eHL, eHealth literacy; LPA, light physical activity; MVPA, moderate-to-vigorous physical activity; SB, sedentary behavior.

### 3.4 Moderating role of self-efficacy

**Figure 2:**
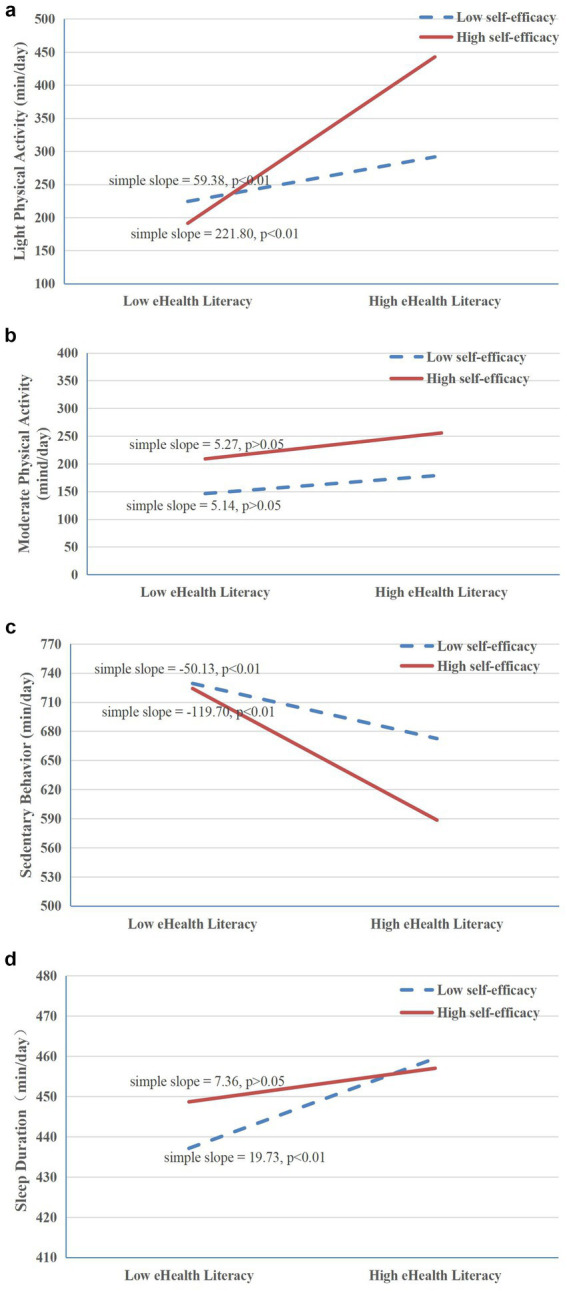
**(a)** Interaction between eHL and self-efficacy on LPA. **(b)** Interaction between eHL and self-efficacy on MVPA. **(c)** Interaction between eHL and self-efficacy on SB. **(d)** Interaction between eHL and self-efficacy on sleep duration. eHL, eHealth literacy; LPA, light physical activity; MVPA, moderate-to-vigorous physical activity; SB, sedentary behavior.

Moderation analyses revealed that self-efficacy significantly moderated the associations between eHL and LPA (*F* = 76.82, *p* < 0.001), sleep duration (*F* = 4.74, *p* < 0.001), and SB (*F* = 75.07, *p* < 0.001). The interaction between eHL and self-efficacy for MVPA was not statistically significant (Figure 2b). Simple-slope probes indicated that at high self-efficacy levels (Mean + 1 standard deviation), greater eHL was associated with more LPA and less SB, whereas these associations were attenuated at low levels of self-efficacy (Mean − 1 standard deviation). These interaction patterns were illustrated in Figures 2a,c. For sleep duration, participants with higher self-efficacy showed longer sleep duration at lower levels of eHL. However, as eHL increased, the difference in sleep duration between high and low self-efficacy groups narrowed, resulting in a modest interaction effect (Figure 2d). Although statistically significant, the effect for was small and should be interpreted with caution. In summary, the moderation findings indicate that the association between eHL and several movement outcomes vary by self-efficacy, although the magnitude of these interaction effects, particularly for sleep, appears small.

### 3.5 Sensitivity analyses

Sensitivity analyses were conducted by randomly excluding 5% of participants (*n* = 525). The results were materially unchanged: self-efficacy showed statistically significant indirect effects for LPA, MVPA, and SB, but not for sleep duration (Supplementary Table S1). The moderation findings were also consistent with the primary analyses (Appendix 1).

## 4 Discussion

In this multi-city sample of Chinese older adults, higher eHL was associated with higher LPA and MVPA and lower SB. Self-efficacy emerged as both a partial mediator and a moderator of these associations for LPA and SB, whereas these effects were weaker or absent for sleep duration and MVPA. Importantly, although several associations reached statistical significance, both the direct and indirect effects was modest in magnitude, underscoring the need for cautious interpretation of their practical significance. Overall, these findings suggest that eHL is related to more favorable 24-HMB profiles in older adults, but these associations appear contingent on psychological resources such as self-efficacy and may be stronger for lower-intensity, routine-based behaviors than for sleep or higher-intensity activity.

### 4.1 Interpretation of associations and psychological pathways

From a Social Cognitive Theory (SCT) perspective, health-related behaviors are shaped by reciprocal interactions among personal factors, behavioral capabilities, and environmental influences, with self-efficacy representing a central determinant of behavior initiation and maintenance (12). Within this framework, eHL may facilitate engagement in health-related behaviors by enhancing individuals’ capacity to access, appraise, and apply digital health information, whereas self-efficacy reflects confidence in translating such knowledge into action.

In the present study, self-efficacy partially explained the associations between eHL and both LPA and SB, suggesting that older adults with higher self-efficacy may be more likely to act on digital health information in their daily routines. However, the indirect effects observed were small in magnitude, indicating that self-efficacy accounts for only a limited proportion of the association between eHL and movement behaviors. These findings align with prior work showing that eHL and related psychological resources typically exert modest effects in observational designs (31–35), particularly among older adults whose behaviors are also shaped by physical capacity, health status, and environmental constraints (36, 37).

The moderation findings further indicate that eHL was more strongly associated with LPA and SB among individuals with higher self-efficacy, whereas these associations were attenuated at lower levels of self-efficacy. This pattern was not observed for MVPA, which is consistent with evidence that higher-intensity PA in later life is more strongly constrained by physiological limitations and contextual barriers than by informational or motivational factors alone (36, 37). Collectively, these results suggest that eHL may be more readily translated into low-threshold behaviors, such as light movement and interrupting sitting time, particularly among individuals with greater confidence in their ability to change behavior.

### 4.2 Sleep duration: limited mediation and modest moderation

In contrast to PA and SB, the role of self-efficacy in the association between eHL and sleep duration was limited. Although eHL showed a small but statistically significant total association with sleep duration, neither mediation through self-efficacy nor strong moderation effects were supported. The observed interaction between eHL and self-efficacy for sleep was statistically significant but small in magnitude, indicating a modest effect that should be interpreted with caution.

These findings are consistent with the mixed literature on digital health resources and sleep in older adults. While some studies report beneficial effects of sleep-related digital information or interventions (e.g., sleep hygiene education, relaxation tools) on sleep outcomes (38, 39), others suggest that technology use, particularly in the evening, may disrupt sleep via increased cognitive arousal or light exposure (40, 41). Such heterogeneity likely reflects differences in content type, timing of use, delivery mode, and individual self-regulation.

Within this context, the present findings suggest that eHL alone is unlikely to exert a strong or direct influence on sleep duration. Instead, sleep in older adults may be shaped more strongly by physiological factors, long-established routines, and health-related constraints, thereby limiting the extent to which informational resources and self-efficacy translate into meaningful sleep-related change.

### 4.3 Positioning within the existing literature

The present results are broadly consistent with prior studies linking higher eHL to greater PA and lower SB among older adults (31–35). However, by examining these associations within an integrated 24-HMB framework and by jointly testing mediation and moderation processes, this study extends existing work by clarifying how and under what conditions eHL may relate to different behavioral domains.

Importantly, the modest effect sizes observed align with prior research suggesting that eHL and related psychosocial resources represent only one component within a broader constellation of determinants influencing movement behaviors in later life (31–37). Compared with intervention studies that directly manipulate digital tools or behavior-change techniques, the present observational findings reflect naturally occurring variability in literacy and self-efficacy, which may partly explain the relatively small magnitude of effects. Together, these results highlight the importance of avoiding overgeneralization and of situating eHL as a complementary, rather than dominant, determinant of movement behavior in older adults.

### 4.4 Implications, limitations, and future directions

From a practical perspective, the findings suggest that interventions aiming to promote healthy movement behaviors in older adults may benefit from addressing both eHL and self-efficacy. Importantly, the associations were more consistent for low-threshold, routine-based behaviors (particularly LPA and SB), indicating that eHealth-enabled strategies may be most impactful when prioritizing incremental movement across the day and interrupting sitting time, rather than focusing exclusively on vigorous activity. In addition, given the moderation results, support strategies may need to be tailored by self-efficacy level, as individuals with lower self-efficacy appeared less likely to translate eHL into behavioral differences. These implications align with contemporary public health guidance and integrated 24-HMB frameworks, which emphasize feasible, day-long behavior change approaches for older adults (42, 43).

Several limitations warrant explicit consideration. First, reliance on an online survey likely excluded older adults with very low digital skills, potentially introducing selection bias and limiting generalizability (e.g., potential overestimation of eHL and positive eHealth attitudes in the analytic sample). Moreover, the sample was restricted to urban, community-dwelling adults aged 60–74 years who could independently use digital devices. Therefore, the findings may not generalize to older-old adults, rural settings, or individuals with minimal digital access. Second, although accelerometry provides objective estimates of PA and SB, the three-day wear protocol may not fully capture habitual patterns or week-to-week variability. This concern is particularly relevant in older populations, where device-based evidence shows substantial heterogeneity and inconsistent findings, especially for SB outcomes, underscoring the need for cautious interpretation of accelerometer-derived estimates (30). Third, the cross-sectional design precludes causal inference and limits conclusions regarding temporal ordering among eHL, self-efficacy, and movement behaviors. Finally, residual confounding remains possible because several potentially relevant factors (e.g., health conditions, functional status, prior digital experience, economic status, and cultural contexts) were not available in the current dataset and should be incorporated in future research.

Future research should prioritize both digital inclusion and methodological rigor. Mixed-method recruitment (e.g., combining online approaches with telephone- or paper-based protocols) and probability-based or multisite sampling would further improve representativeness across the spectrum of eHL. Extending accelerometer wear periods and incorporating device- and person-specific compliance strategies may enhance measurement stability and better reflect habitual behavior. Longitudinal or experimental designs are needed to test causal pathways and to clarify temporal dynamics between eHL, self-efficacy, and 24-HMB. Finally, incorporating a broader range of contextual and health-related covariates, such as health status, functional limitations, medication use, and environmental factors, may help identify the conditions under which eHL contributes to meaningful behavior change in older adults.

## 5 Conclusion

In a multi-city sample of Chinese older adults, higher eHL was associated with a more favorable 24-HMB profile, particularly higher light-intensity PA and lower sedentary time, whereas associations with sleep duration were weaker. Self-efficacy showed modest mediating and moderating roles in several associations, suggesting that the behavioral relevance of eHL may depend partly on individuals’ confidence in applying health information in daily life. Given the cross-sectional design and small effect sizes, these findings should not be interpreted as causal and should be interpreted cautiously. By integrating the 24-HMB framework with a theory-informed test of psychological pathways, this study extends prior work beyond descriptive links between eHL and single behaviors. Future research should use longitudinal or experimental designs, more representative sampling, and improved sleep assessment, and should incorporate key health and contextual covariates to clarify causal directions and identify for whom, and under what conditions, eHL supports meaningful behavior change.

## Data Availability

The original contributions presented in the study are included in the article/Supplementary material, further inquiries can be directed to the corresponding authors.
