# Supplementary material for: Associations between eHealth literacy and 24-hour movement behaviors in older adults: the mediating and moderating roles of self-efficacy
Source: Front Med (Lausanne). 2026 Mar 11;13:1746861. doi: 10.3389/fmed.2026.1746861 (PMC13013294; doi:10.3389/fmed.2026.1746861)
Supplement: Supplementary file 1 [file Data_Sheet_1.PDF]

1. 性别：☐男 ☐女

2. 年龄：________ 岁

3. 婚姻状况：（已婚/离异/丧偶/单身）

4. 受教育程度：

☐未受过教育 ☐未读完小学但能听说读写 ☐小学毕业

☐初高中毕业 ☐大专及以上学历

5. 您现在和谁住在一起：☐和他人居住（如：配偶、子女） ☐单独居住

6. 您的身高是________米；体重是________公斤

7. 您如果愿意参加我们后续的调查，请留下您的姓和联络电话：

姓：__________；电话：_________

A. 电子健康素养

**对于以下陈述，请选择最符合您情况的数字（请逐一评价）**	**非常不同意**				**非常同意**
1. 我知道应该选择哪种网络健康工具满足我的健康需求(如查阅药物说明、进行健康咨询、制定减肥计划等不同方面的健康需求)。	1	2	3	4	5
2. 我能够判断网络健康工具是否可信。(如：判断网络健康咨询等)	1	2	3	4	5
3. 我会去通过网络获取健康信息（如：医疗信息,运动保健信息等)。	1	2	3	4	5
4. 我知道网上哪里可以找到有帮助的健康资源。	1	2	3	4	5
5. 在网上与他人交流时,我能够十分清晰地表达与健康相关的担忧。	1	2	3	4	5
6. 回答网络平台上的健康求助时，我能够给出负责任的回答(即，我的回答不会误导他人,也不会伤 害自己的信息安全)。	1	2	3	4	5
7. 我能够判断网上的信息是否带有商业利益(如:提供该信息的人 是为了销售某一产品)。	1	2	3	4	5

8. 使用网络健康工具时,我会去维护信息的原创性(如:不盗用他人发布的原创内容,举报剽窃行为等)。	1	2	3	4	5
9. 使用网络查询健康信息时,我会去查看作者的资历证书与所属机构。	1	2	3	4	5
10. 使用网络查询健康信息时,我会去查看网页的拥有者。	1	2	3	4	5
11. 使用网络查询健康信息时,我会去查看网页最近一次的更新日期。	1	2	3	4	5
12. 使用网络查询健康信息时,我会去查看是否有其他出版物或网络资源确认过此信息。	1	2	3	4	5
13. 我知道如何从多个渠道查看网络健康信息。	1	2	3	4	5
14. 即使是信任的人告诉我的健康信息,我也会上网去查阅它。	1	2	3	4	5
15. 我知道如何使用网络健康工具对我的健康行为进行记录。(如:使用智能手表记录健康信息)	1	2	3	4	5
16. 我知道如何利用网络健康工具上的记录为我的日常健康管理提供参考。	1	2	3	4	5
17. 我知道如何利用网络健康工具对我的健康行为进行追踪(如:从以往的使用记录中了解自身运动频率、体重等身体指针的变化曲线)。	1	2	3	4	5
18. 若有需要,我能够坚持使用某一网络健康工具(如手机软件,体脂秤或手环等)。	1	2	3	4	5
19. 若有需要,我能够有计划地使用网络健康工具。	1	2	3	4	5
20. 使用网络健康工具的过程中,我能够根据实际情况调整自己的使用频率、强度与方式。	1	2	3	4	5
21. 我知道如何在网络健康工具上发	1	2	3	4	5

布和分享自己的健康行为(如：将运动轨迹分享到健康记录软件或朋友圈)。					
22. 我知道如何使用社交软件上的运动功能(如微信运动)与他人进行互动(如点赞、助力等）。	1	2	3	4	5
23. 我会将我在网络健康工具上所关注的人作为目标,去效仿和赶超他(她)。	1	2	3	4	5
24. 我会去尝试网上与健康有关的提议，并控制其中的风险(如:当发烧时，听取网络提供的健康意见，同时根据自身情况，有选择性地控制药物剂量，避免过度服用)。	1	2	3	4	5

B. 自我效能

**我确信自己能够进行健康的生活方式，（如定期参加体育活动、保持充足睡眠）**	**非常不同意**				**非常同意**
1. 即使很难，我仍相信自己可以做到	1	2	3	4	5
**我确信自己能够长期坚持健康的生活方式（如坚持参加体育活动、保持充足睡眠）**	**非常不同意**				**非常同意**
2. 即使我需要花很长的时间适应这样的生活方式，我仍相信自己能够坚持	1	2	3	4	5
3. 即使我在过程中遇到一些困难(例如:没有时间锻炼、天气场地原因等)，我仍相信自己能够克服困难，并长期坚持	1	2	3	4	5
**我确信自己能够在中断后，重新开始健康的生活方式，(如定期参加体育活动、保持充足睡眠)**	**非常不同意**				**非常同意**
4. 如：即使我有几次改变了计划，我仍相信可以再次开始	1	2	3	4	5
5. 如:即使有几天没有进行健康的生活，比如因为生病或其他原因中断了体育锻炼。当身体恢复后，我仍相信自己可以重新做到	1	2	3	4	5

睡眠日志

请根据您的实际情况，回答下列问题：

1. 早上___________点起床（24小时制，如6:30））
2. 晚上___________点睡觉（24小时制，如21:30）
3. 晚上实际睡眠_____________小时（不等于卧床时间，24小时制）
