# Supplementary material for: Associations between eHealth literacy and 24-hour movement behaviors in older adults: the mediating and moderating roles of self-efficacy
Source: Front Med (Lausanne). 2026 Mar 11;13:1746861. doi: 10.3389/fmed.2026.1746861 (PMC13013294; doi:10.3389/fmed.2026.1746861)
Supplement: Supplementary file 2 [file Data_Sheet_2.PDF]

Questionnaire (English Translation)

Demographic Information

1. Sex: □ Male □ Female

2. Age: ________ years

3. Marital status: □ Married □ Divorced □ Widowed □ Single

4. Education level:

□ No formal education

□ Did not complete primary school but can listen/speak/read/write

□ Primary school graduate

□ Middle/high school graduate

□ College or above

5. Current living arrangement: □ Living with others (e.g., spouse, children) □ Living alone

6. Height: ________ m Weight: ________ kg

7. If you are willing to participate in follow-up surveys, please leave your family name and contact number:

Family name: __________ Phone: __________

A. eHealth Literacy

For each statement below, please choose the number that best matches your situation.

1. I know which online health tools to choose to meet my health needs (e.g., reading medication instructions, seeking health consultations, making a weight-loss plan).				
1<br>Strongly disagree	2	3	4	5<br>Strongly agree
2. I can judge whether an online health tool is trustworthy (e.g., online health consultations).				
1<br>Strongly disagree	2	3	4	5<br>Strongly agree
3. I obtain health information through the internet (e.g., medical information, exercise/health-care information).				
1<br>Strongly disagree	2	3	4	5<br>Strongly agree

4. I know where to find helpful health resources online.				
1<br>Strongly disagree	2	3	4	5<br>Strongly agree
5. When communicating with others online, I can express my health-related concerns very clearly.				
1<br>Strongly disagree	2	3	4	5<br>Strongly agree
6. When responding to others' health-related help requests on online platforms, I can provide responsible answers (i.e., my response will not mislead others and will not compromise my own information security).				
1<br>Strongly disagree	2	3	4	5<br>Strongly agree
7. I can judge whether online information involves commercial interests (e.g., the provider is trying to sell a product).				
1<br>Strongly disagree	2	3	4	5<br>Strongly agree
8. When using online health tools, I help maintain originality of information (e.g., do not plagiarize others' original content; report plagiarism).				
1<br>Strongly disagree	2	3	4	5<br>Strongly agree
9. When searching for health information online, I check the author's credentials and affiliated institution.				
1<br>Strongly disagree	2	3	4	5<br>Strongly agree
10. When searching for health information online, I check who owns the website.				
1<br>Strongly disagree	2	3	4	5<br>Strongly agree
11. When searching for health information online, I check the date when the webpage was last updated.				
1<br>Strongly disagree	2	3	4	5<br>Strongly agree
12. When searching for health information online, I check whether other publications or online resources have confirmed the information.				
1<br>Strongly disagree	2	3	4	5<br>Strongly agree
13. I know how to consult online health information from multiple channels.				
1<br>Strongly disagree	2	3	4	5<br>Strongly agree

14. Even if health information is told to me by someone I trust, I still look it up online.				
1<br>Strongly disagree	2	3	4	5<br>Strongly agree
15. I know how to use online health tools to record my health behaviors (e.g., using a smartwatch to record health information).				
1<br>Strongly disagree	2	3	4	5<br>Strongly agree
16. I know how to use the records in online health tools as references for my daily health management.				
1<br>Strongly disagree	2	3	4	5<br>Strongly agree
17. I know how to use online health tools to track my health behaviors (e.g., using past records to view changes in exercise frequency, weight, and other indicators).				
1<br>Strongly disagree	2	3	4	5<br>Strongly agree
18. If needed, I can keep using a particular online health tool (e.g., mobile apps, smart body-fat scales, or wristbands).				
1<br>Strongly disagree	2	3	4	5<br>Strongly agree
19. If needed, I can use online health tools in a planned way.				
1<br>Strongly disagree	2	3	4	5<br>Strongly agree
20. During use of online health tools, I can adjust my frequency, intensity, and methods of use according to actual circumstances.				
1<br>Strongly disagree	2	3	4	5<br>Strongly agree
21. I know how to post and share my health behaviors on online health tools (e.g., sharing exercise routes to a health-record app or social media).				
1<br>Strongly disagree	2	3	4	5<br>Strongly agree
22. I know how to use the exercise functions on social apps (e.g., WeChat Step Count) to interact with others (e.g., likes, “boosts”).				
1<br>Strongly disagree	2	3	4	5<br>Strongly agree
23. I take people I follow on online health tools as targets and try to learn from and catch up with them.				
1<br>Strongly disagree	2	3	4	5<br>Strongly agree

24. I try health-related suggestions online and manage potential risks (e.g., when having a fever, follow online advice while adjusting medication dosage prudently to avoid overuse).				
1<br>Strongly disagree	2	3	4	5<br>Strongly agree

B. Self-Efficacy

Each item begins with: “I am confident that…” Please choose the number that best matches your situation.

1. I can maintain a healthy lifestyle (e.g., engage in regular physical activity and get enough sleep), even if it is difficult.				
1<br>Strongly disagree	2	3	4	5<br>Strongly agree

2. I can maintain a healthy lifestyle over the long term (e.g., continue physical activity and get enough sleep), even if it takes a long time to adapt.				
1<br>Strongly disagree	2	3	4	5<br>Strongly agree

3. I can maintain a healthy lifestyle over the long term even if I encounter difficulties (e.g., lack of time, weather/venue constraints).				
1<br>Strongly disagree	2	3	4	5<br>Strongly agree

4. I can restart a healthy lifestyle after an interruption, even if I have changed my plan a few times.				
1<br>Strongly disagree	2	3	4	5<br>Strongly agree

5. I can resume a healthy lifestyle after a few unhealthy days (e.g., physical activity was interrupted due to illness), once I recover.				
1<br>Strongly disagree	2	3	4	5<br>Strongly agree

Daily Sleep Log

Please complete the following based on your actual situation:

1. Wake-up time in the morning: __________ (24-hour format, e.g., 06:30)

2. Bedtime at night: __________ (24-hour format, e.g., 21:30)

3. Actual sleep duration at night: __________ hours (not the same as time in bed)
