# Supplementary material for: Associations between eHealth literacy and 24-hour movement behaviors in older adults: the mediating and moderating roles of self-efficacy
Source: Front Med (Lausanne). 2026 Mar 11;13:1746861. doi: 10.3389/fmed.2026.1746861 (PMC13013294; doi:10.3389/fmed.2026.1746861)
Supplement: Supplementary file 3 [file Data_Sheet_3.PDF]

Appendix 1.

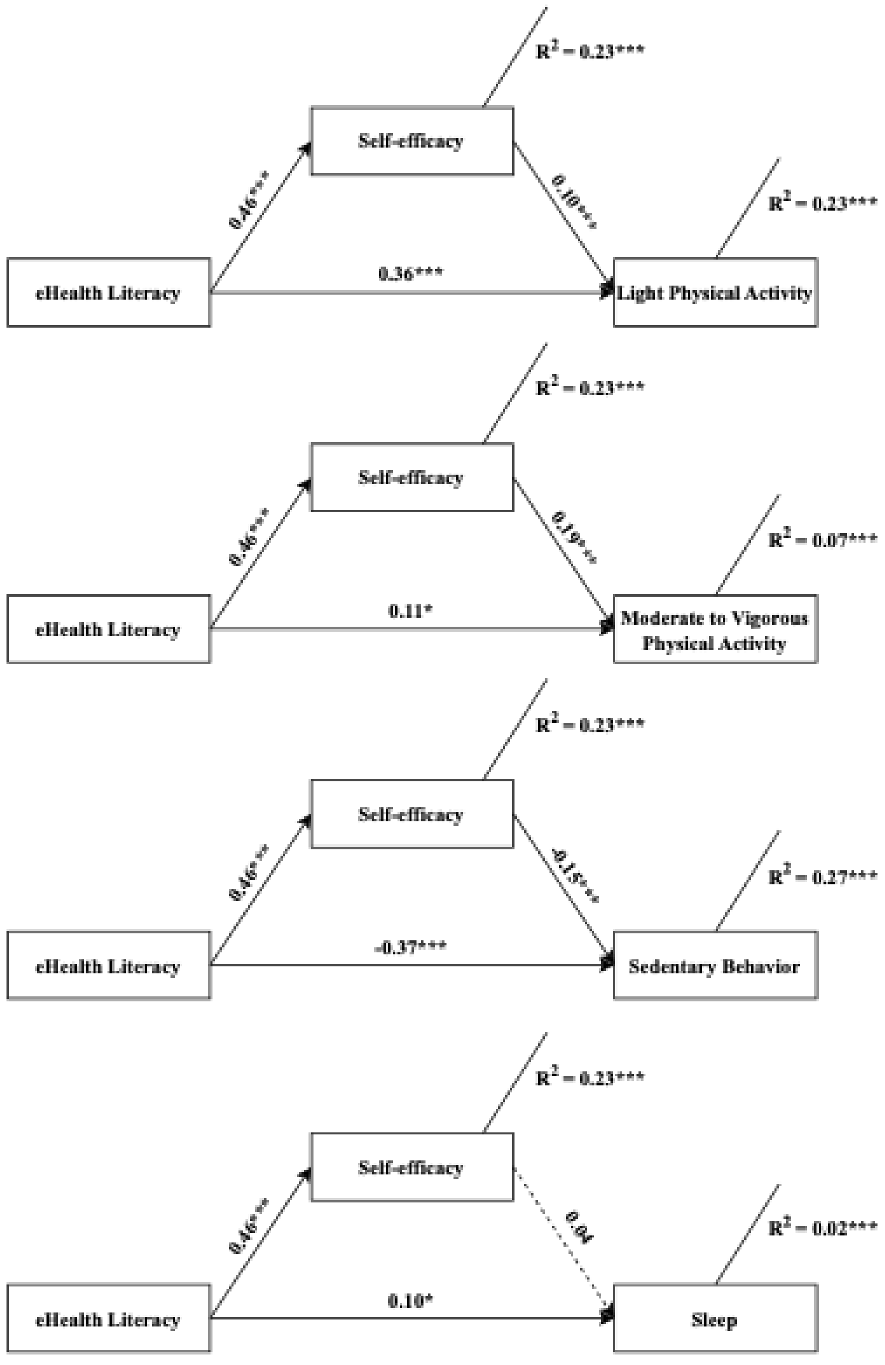

Figure 1. Mediation models illustrating the indirect effects of eHL on LPA, MVPA, SB, and sleep through self-efficacy.

Note: Age, sex, and educational level were controlled as covariates in all mediation models. Solid lines represent significant paths, whereas dashed lines represent non-significant paths. Values reported are standardized path coefficients. *$p < .05$, ** $p < .01$, *** $p < .001$. eHL, eHealth literacy. LPA, light physical activity. MVPA, moderate-to-vigorous physical activity. SB, sedentary behavior.

Table 1 Total, direct, and indirect effects of eHL on LPA, MVPA, SB, and sleep through self-efficacy (n = 525).

**variable**	*Est* (95% CI)
**Total effect**	
eHL → LPA	0.41 ***
eHL → MVPA	0.20***
eHL → SB	-0.43***
eHL → Sleep	0.12**
**Indirect effects**	
eHL → self-efficacy → LPA	0.04 (0.00, 0.08)
eHL → self-efficacy → MVPA	0.09 (0.04, 0.14)
eHL → self-efficacy → SB	-0.07 (-0.11, -0.03)
eHL → Self-efficacy → Sleep	0.02 (-0.03, 0.07)
**Direct effect**	
eHL → LPA	0.41 *
eHL → MVPA	0.11*
eHL → SB	-0.37***
eHL → Sleep	0.10

Note: Age, sex, and educational level were controlled as covariates in all models; *Est* represents the estimated standardized effect; 95% *CI* denotes the 95% confidence interval estimated using bias-corrected bootstrap procedures with 5,000 resamples; *p< 0.05, **p< 0.01, ***p< 0.001. eHL, eHealth literacy. LPA, light physical activity. MVPA, moderate-to-vigorous physical activity. SB, sedentary behavior.

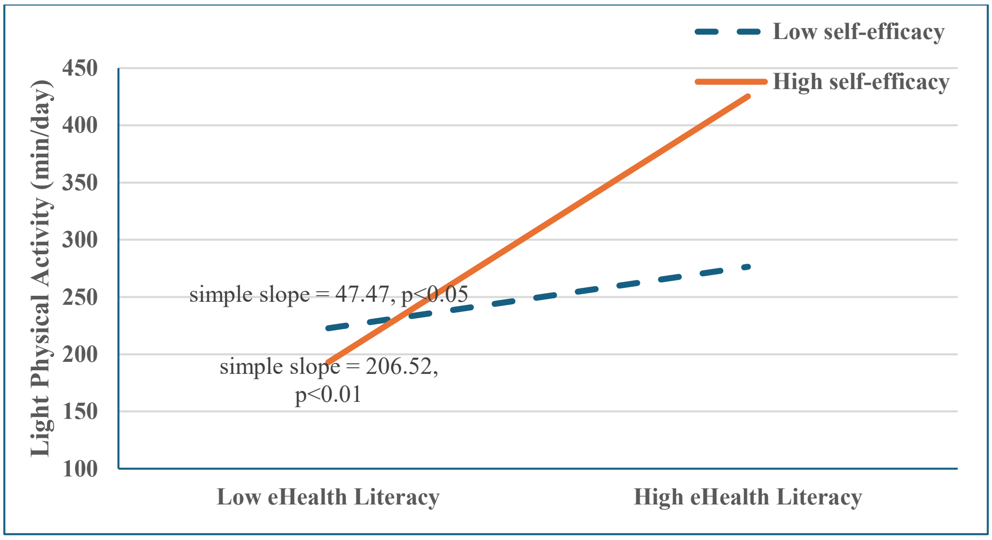

Figure 2a Interaction between eHL and self-efficacy on LPA

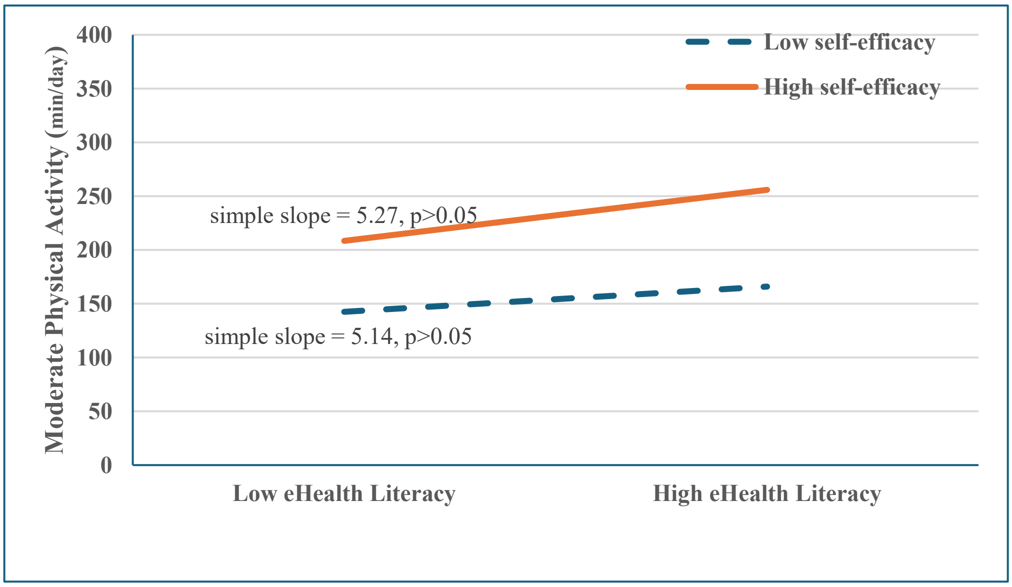

Figure 2b Interaction between eHL and self-efficacy on MVPA

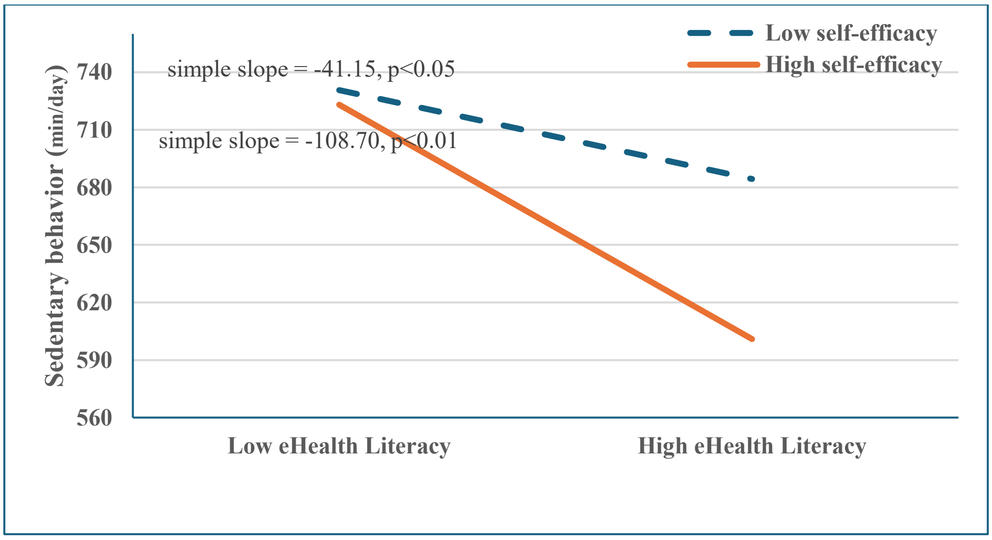

Figure 2c Interaction between eHL and self-efficacy on SB

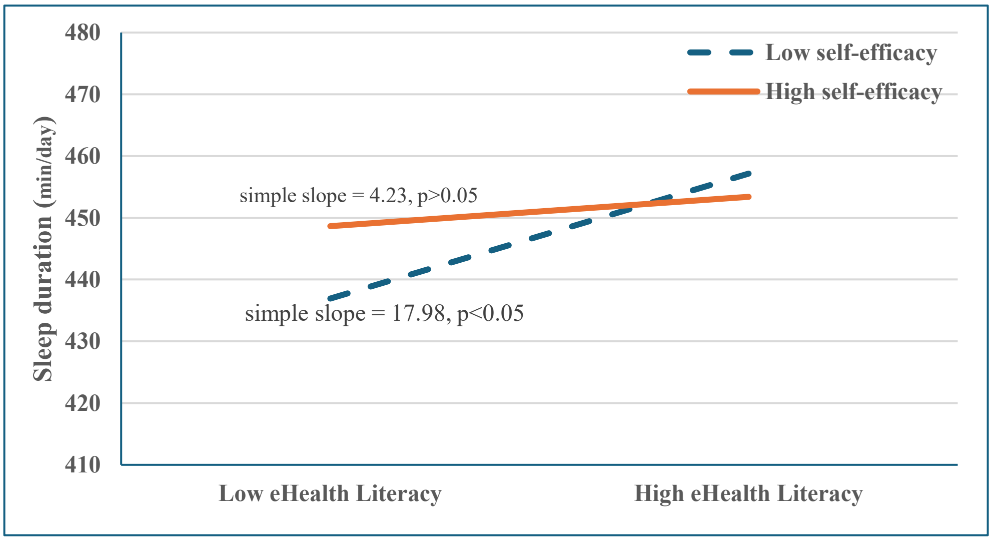

Figure 2d Interaction between eHL and self-efficacy on sleep duration

Note: eHL, eHealth literacy. LPA, light physical activity. MVPA, moderate-to-vigorous physical activity. SB, sedentary behavior.
